# Sustainable Community Engaged Exercise and Physical Activity for Underrepresented Older Adults

**DOI:** 10.1093/geroni/igaf122.926

**Published:** 2025-12-31

**Authors:** Rebecca Lassell, Laura Baehr, Brian Andonian

## Abstract

This symposium aims to showcase three approaches to community-engaged research for exercise and physical activity intervention development and implementation for underrepresented older adults. Presenters will provide a how-to guide with examples of recruitment and study materials and community engagement strategies as well as lessons learned and recommendations for early career investigators. Four presenters will provide examples from their research. Presentations will include a co-design process to identify community challenges and solutions to design a nature lifestyle intervention for Chinese American older adults, a community-engaged fitness facility recreational needs assessment to enhance participation by older adults; and the implementation of a peer-supported intervention for Latinx older adults with Parkinson’s. This symposium will cover 1) How to build trust and relationships communities with two examples from institutions with varying levels of infrastructure and resources 2) different methodological approaches to understanding community needs ranging from needs assessments to a six-step co-design process, 3) strategies to engage underrepresented older adults in exercise and physical activity intervention design and implementation, and 4) new learnings and a how-to guide from early career investigators to start and implement exercise and physical activity interventions in the NIH Stage Model Stages 0-1. Fitness, Exercise and Wellness Interest Group Sponsored Symposium

